# Distinct Modulation of Event-Related Potentials during Motor Preparation in Patients with Motor Conversion Disorder

**DOI:** 10.1371/journal.pone.0062539

**Published:** 2013-04-23

**Authors:** Rebekah L. Blakemore, Brian I. Hyland, Graeme D. Hammond-Tooke, J. Greg Anson

**Affiliations:** 1 School of Physical Education, University of Otago, Dunedin, New Zealand; 2 Brain Health Research Centre, University of Otago, Dunedin, New Zealand; 3 Department of Physiology, University of Otago, Dunedin, New Zealand; 4 Department of Medicine, University of Otago, Dunedin, New Zealand; 5 Department of Sport and Exercise Science, and Centre for Brain Research, University of Auckland, Auckland, New Zealand; University of Toronto, Canada

## Abstract

**Objective:**

Conversion paresis patients and healthy people feigning weakness both exhibit weak voluntary movement without detectable neuropathology. Uniquely, conversion patients lack a sense of conscious awareness of the origin of their impairment. We investigated whether conversion paresis patients show distinct electroencephalographic (EEG) markers associated with their unconscious movement deficits.

**Methods:**

Six unilateral upper limb conversion paresis patients, 12 feigning participants asked to mimic weakness and 12 control participants performed a precued reaction time task, requiring movements of either hand, depending on precue information. Performance measures (force, reaction and movement time), and event-related EEG potentials (ERP) were compared, between groups and across hands or hemisphere, using linear mixed models.

**Results:**

Feigners generated the same inter-hand difference in reaction and movement time as expressed by patients, even though no specific targets were set nor feedback given on these measures. We found novel ERP signatures specific to patients. When the symptomatic hand was precued, the P3 ERP component accompanying the precue was dramatically larger in patients than in feigning participants. Additionally, in patients the earlier N1 ERP component was diminished when the precue signalled either the symptomatic or asymptomatic hand.

**Conclusions:**

These results are consistent with previous suggestions that lack of awareness of the origin of their symptoms in conversion disorder patients may result from suppression of brain activity normally related to self-agency. In patients the diminished N1 to all precues is consistent with a generalised reduction in cognitive processing of movement-related precues. The P3 enhancement in patients is unlikely to simply reflect changes required for generation of impaired movements, because it was not seen in feigners showing the same behavioural deficits. Rather, this P3 enhancement in patients may represent a neural biomarker of unconscious processes, including additional emotional loading, related to active suppression of brain circuits involved in the attribution of self-agency.

## Introduction

Conversion disorder is a poorly understood syndrome, thought to be triggered by psychological stressors such as trauma or conflict, in which patients present with neurological symptoms that cannot be explained by any underlying neuropathology [1], [2]_ENREF_1_ENREF_2. Conversion disorder has many presentations, including epileptiform, sensory and motor manifestations. The prevalence of all Conversion disorder subtypes are reported to range from 0.01–0.3% in the general population [1]. Motor disorders include tremor, *paralysis* (i.e., a complete inability to move the affected part), and *paresis*, in which patients demonstrate unexplained muscle weakness during intentional movement. In both conversion paralysis and paresis, unconscious movements (e.g., automatic postural adjustments) and reflexes involving the symptomatic muscle groups remain present [1] and there is often inconsistency of symptoms during clinical observation [3], indicating the neuromotor apparatus for movement of the symptomatic limb is intact.

Previous studies have used functional imaging to investigate neural correlates of conversion paralysis and have demonstrated altered blood flow in the cerebral cortex while the patients were at rest [4], [5] or during movement attempts[6]–[9]. Various changes in cortical blood flow and somatosensory evoked potentials have also been described in conversion patients with dominant sensory symptoms [10]–[13]. However to date, no data are available about direct time-dependent changes in cortical processing associated with preparation for movement in conversion paresis. Significantly, of the studies examining patients with conversion paresis or paralysis, many have been single-subject designs, with few studies including a patient cohort greater than two (e.g., Stone et al., *n* = 4 [8], Vuilleumier et al., *n* = 7 [5]).

Here, we investigated in six patients, all presenting with unilateral upper limb conversion paresis, changes in behaviour and in the amplitude of ERPs (specifically P1, N1, P3 and the contingent negative variation [CNV]) triggered by visual stimuli enabling preparation of movement by either hand, during preparation of a reaction time (RT) task. Comparison with matched controls who consciously feigned weakness revealed several neural correlates unique to the patients. These appear to index specific neural processes associated with altered awareness of their state of consciousness in the generation of impaired movement.

## Materials and Methods

### Participant Recruitment

To examine the neurophysiological correlates of unconscious (conversion) versus intentional (feigned) paresis, we tested six conversion disorder patients (4 female, mean age 57±4 years; mean symptom duration 18±4 months) with unilateral (5/6 left) upper limb weakness according to the Diagnostic and Statistical Manual of Mental Disorders (DSM-IV) criteria [1] (Table 1). For every patient, age- and sex-matched healthy volunteers were randomly assigned to either a standard control group (referred to as “controls”) or a feigning group (“feigners”). Feigners were instructed to mimic weakness by imagining that their left arm, hand and fingers had become so weak they would find it very difficult, but not impossible, to move the limb.

**Table 1:** Characteristics of participants.

Participants	Gender	Age (years)	Duration of symptoms (months)	aSensory deficits	Symptomatic hand	bMVC symptomatic hand (N)	MVC asymptomatic hand (N)	cPsychiatric comorbidity (DSM-IV)
Patient 1	female	53	24	+	left	10.30	17.46	300.23
Patient 2	female	56	40	++	left	1.32	30.25	none
Patient 3	female	58	11	+++	right	5.63	13.66	296.23, 300.02
Patient 4	female	51	7	none	left	2.22	7.54	30.29N
Patient 5	male	71	24	++	left	4.72	21.89	none
Patient 6	male	52	1	none	left	15.32	19.56	none
Feigners (*n* = 12)	8 female	54±2	–	–	12 left	8.94±6.69	40.17±12.5	–
Controls (*n* = 12)	8 female	54±4	–	–	–	40.50±12.01	48.28±11.97	–

a Sensory deficits in the symptomatic limb:+small sensory deficits (some numbness on the symptomatic side);++moderate sensory deficits (some numbness on symptomatic side plus decreased sensation for light touch, vibration and temperature);+++severe sensory deficits (some numbness, tingling on symptomatic side, decreased sensation for light touch, vibration and temperature plus presence of vertigo, dizziness).

b MVC force values for the feigners and controls for ‘symptomatic’ and ‘asymptomatic’ hand columns correspond to the left and right hands, respectively.

c Diagnoses present within 12 months prior to testing, as assessed by the Composite International Diagnostic Interview (CIDI-Auto v2.1) [43]. DSM-IV categories: 300.23 social phobia; 296.23 major depressive disorder, single episode, severe without psychotic features; 300.02 generalised anxiety disorder; 300.29N specific phobia, natural environment type.

Clinical details of each patient including individual force data for the maximum voluntary contraction (MVC) task, and group details of the feigners and controls including mean (± SD) force data for the MVC task.

No patients had any history of neuropathology, and all underwent a full neurological examination by a neurologist, and neuroimaging to rule out current organic disease. Patients were excluded if they had affected vision or speech, or pain in their symptomatic limb during task performance. Patients were not excluded if mild somatic sensory deficits were present. Patients were medication free for 10 hours prior to the experimental session. Healthy volunteers reported no mental disorder in the past 12 months, and no history of neurological disorder. All participants were right handed [14], and had normal hearing and normal or corrected-to normal vision.

### Ethics Statement

We conducted the study according to the Declaration of Helsinki. The study was approved by the New Zealand Lower South Regional Human Ethics Committee. All participants provided written informed consent for the collection of data and subsequent analysis, after receiving written and verbal information about the study.

### Task

**Figure 1:**
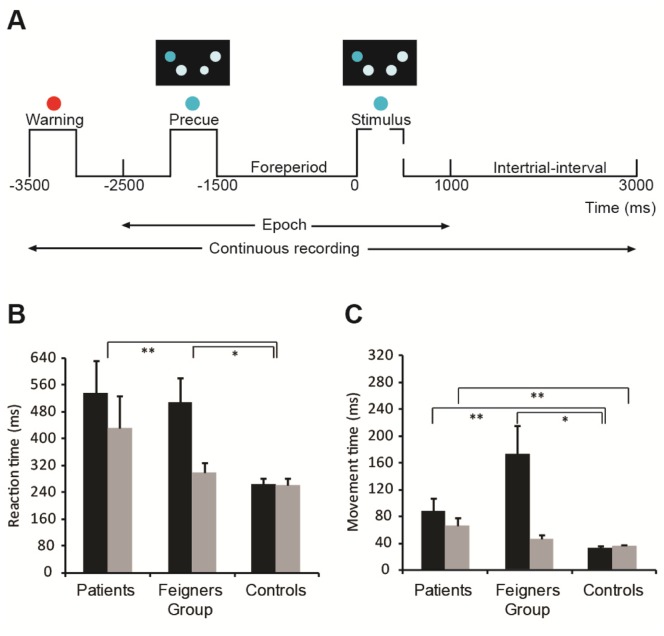
Experimental task and behavioral results. (A) Temporal sequence for each trial. Circles in black rectangle represent response key positions under the middle and index fingers of each hand. In this example, the blue precue symbol signals preparation of the left hand-middle finger. After a foreperiod of 1500 ms, the same blue symbol is illuminated as the imperative stimulus. (B) Mean reaction time (+ SEM) and (C) mean movement time (+ SEM) for the symptomatic (left) hand (black bars) and the asymptomatic (right) hand (grey bars) from patients (*n* = 6), feigners (*n* = 12), and controls (*n* = 12). *P<.05, **P<.01.

Participants performed a finger flexion RT task [15], [16] (Fig. 1A). The response apparatus contained four response keys and a central red warning light. Blue light emitting diodes embedded within each key served as both the precue and imperative “go” stimulus. The middle and index fingers of each hand rested on the proximal end of the corresponding key. On each trial, the key to be pressed remained unknown until presentation of a visual precue (brief illumination of the response key). This provided complete information for participants to prepare the appropriate response during the foreperiod. In the following we use ‘hand’ to refer to pooled data from index and middle fingers of that hand. A force transducer located beneath the proximal end of each key provided continuous force data from which RT and movement time (MT) were measured. RT was calculated as the time between the imperative stimulus and the time at which the applied force exceeded 0.12 N. MT was calculated as the time from this threshold until the end of key displacement (8 mm). Trials were rejected if movement began before or within 100 ms following the “go” signal, the key press was not completed, or the incorrect key was pressed. Outliers (RT>two standard deviations above the mean for each key for each participant) were also removed.

Prior to performing the RT task, the maximum voluntary contraction (MVC) force for each response finger was calculated as the maximum force generated over 2 s from three key presses, with 30 s inter-trial interval, and following 5 warm-up trials of escalating intensity. The maximum values for fingers within hand were averaged to provide an estimate of unilateral force. As expected, patients exhibited distinctly less force (*P = *.024) with their symptomatic hand (Table 1). Feigners produced similar values to patients for their symptomatic hand.

### Electrophysiology

EEG recordings were made bilaterally from frontal (F3, F4), central (C3' and C4'; targeting motor cortex 4 cm to the left and to the right of Cz respectively) [17], parietal (P3, P4) and occipital (O1, O2) sites according to the 10–20 system [18], using sintered Compumedics Quik-Cap™ electrodes and Neuroscan Synamps™. Electrodes were referenced to linked mastoids with a ground electrode at AFz. Eye movements were recorded with vertical and horizontal electrooculography (EOG). All signals were recorded at 1 kHz with bandpass DC-200 Hz and gain 500x. All electrode impedances were maintained below 5 kΩ. DC offsets were corrected online.

Post-recording, a low-pass Butterworth zero phase filter (cut-off frequency 30 Hz, slope 48 dB/octave) and a global DC detrend correction were applied. Eye blink and eye movement artifacts were corrected by subtracting the EOG voltages, multiplied by a channel-independent correction factor, from the EEG voltages_ENREF_24. Before signal averaging, EEG data were epoched (500 ms before precue onset to 1000 ms after imperative stimulus onset), and normalized to baseline calculated from the 500 ms immediately before precue onset. All trials were examined and excessively noisy trials were removed prior to averaging. Mean amplitude of the visual event-related components P1, N1 and P3 were calculated from pre-defined time epochs 100–140 ms, 140–180 ms, and 300–450 ms following precue onset, respectively. Mean CNV amplitude was calculated over the 100 ms immediately prior to the “go” signal.

### Statistical Analyses

Data were analysed using linear mixed models (PASW Statistics 18, SPSS Inc.), allowing inferences to be applied beyond the participant sample [19], [20]. In all analyses, α = .05 and ‘Participant’ was included as a random factor. For behavioral data (RT and MT), the fixed factors were Group (patients, feigners, controls) and Hand (symptomatic, asymptomatic), with Hand as a repeated within-subjects variable. Data from the left hand of controls were compared to the symptomatic hand of patients and feigning participants, while data from the right hand of controls were compared to the asymptomatic hand of patients and feigning participants. For EEG analyses (P1, N1, P3 and CNV amplitude), the factors were Group and Hemisphere, with Hemisphere as a repeated within-subjects variable. Separate analyses were conducted for right and left hand responses, and separately for frontal, central, parietal and occipital electrodes. Additional planned comparisons were also conducted for the P3 and CNV components to directly test the effect of Hand (collapsed over electrodes to avoid confounding by ‘Hemisphere’) on ERP amplitude, for each Group separately. For all analyses, an unstructured model was used for the variance-covariance matrix of the residuals, because the Hurvich and Tsai’s Criterion showed the unstructured heterogeneity model was most appropriate to model the residual matrix. The bonferroni correction was used for all multiple pairwise comparisons.

## Results

### Behaviour

Analysis of RT (Fig. 1B) confirmed the patient and feigning groups initiated movements more slowly with their symptomatic hand compared to controls (Group, F_2,54_ = 8.4, *P* = 0.001, and Hand, F_1,54_ = 5.5, *P* = 0.023; Bonferroni post-hoc tests *P* = 0.001, *P* = 0.012 for patients *vs* control and feigners *vs* controls, respectively). Regression analysis confirmed that RT performance of the symptomatic hand was stable in the patient and feigning groups over the 8 blocks of trials (R^2^ = 0.0212 and 0.0182, respectively). The MT of both patient and feigning groups symptomatic limb was also longer than controls (Fig. 1C; Group by Hand interaction, F_2,54_ = 7.0, *P* = 0.007; Bonferroni post-hoc tests for the symptomatic hand *P* = 0.003 and *P* = 0.012 for patients and feigners *vs* controls, respectively). These results indicate that feigners spontaneously mimicked the RT and MT impairments seen in the symptomatic hand of patients, even though the instructions they were given made no specific reference to slowing movement initiation or performance. This is crucial, because it isolates the difference in conscious awareness between the groups as a key variable, unconfounded by differences in performance.

### Event-related Potentials

We recorded the EEG to measure the sensory ERPs elicited by the visual precue, and the subsequent CNV, associated with preparation for movement [21]. Positive and negative components of the visual ERP within the precue (500 ms) reflect specific aspects of attention and information coding. Here we focus on components at latencies typically labeled as P1, N1 and P3. Data for the patient with a right arm paresis were omitted from EEG analyses to retain consistency of the right hemisphere being contralateral to the symptomatic limb for all remaining patients and feigners, thus n = 5 for EEG analyses of the patient group.

**Figure 2:**
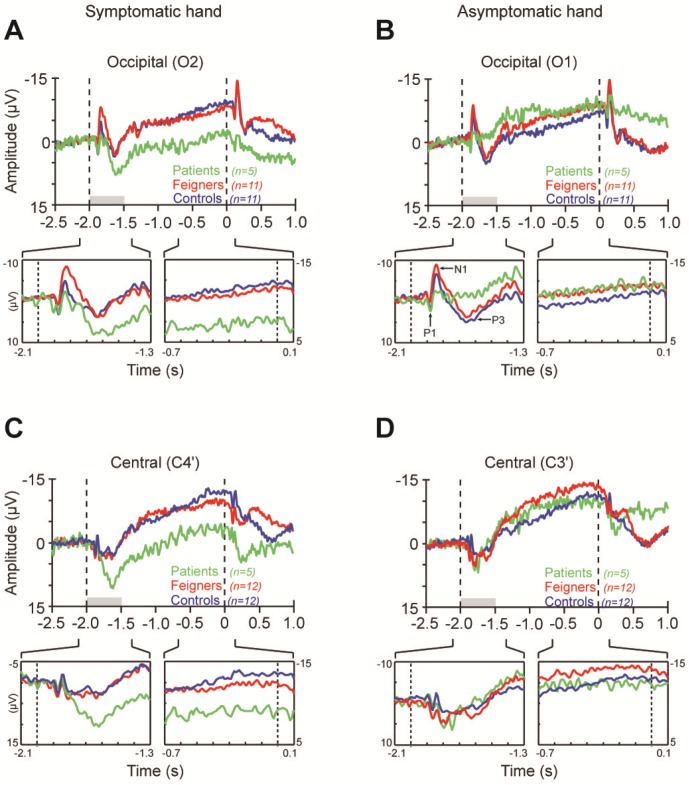
Grand mean EEG waveforms. (A) Symptomatic (left) hand, recording from contralateral occipital cortex. Top plot shows average across entire trial duration, dashed lines at t = −2.0 s and t = 0 s indicate precue and imperative stimulus onsets, grey horizontal bar shows precue duration. Inset panels zoom on precue onset (left; visual ERP) and before stimulus onset (right; terminal CNV). (B) Asymptomatic (right) hand, recording from contralateral occipital cortex. (C) As for A, recording from motor cortex contralateral to the symptomatic hand. (D) As for B, recording from motor cortex contralateral to the asymptomatic hand. Negative upwards in all plots.

The first visual ERP is P1. P1 was observed at occipital electrodes (Fig. 2A,B) as expected [22]. There were no significant Group or Hemisphere effects on amplitude of P1 in response to either the left or right spatially distributed precues that signaled movement of the left or right hand, respectively. Because P1 is known to be modulated by variations in level of spatial attention [23], [24], these non-significant results indicate patients had no deficit of global visual-spatial attention during the earliest sensory processing stages.

**Figure 3:**
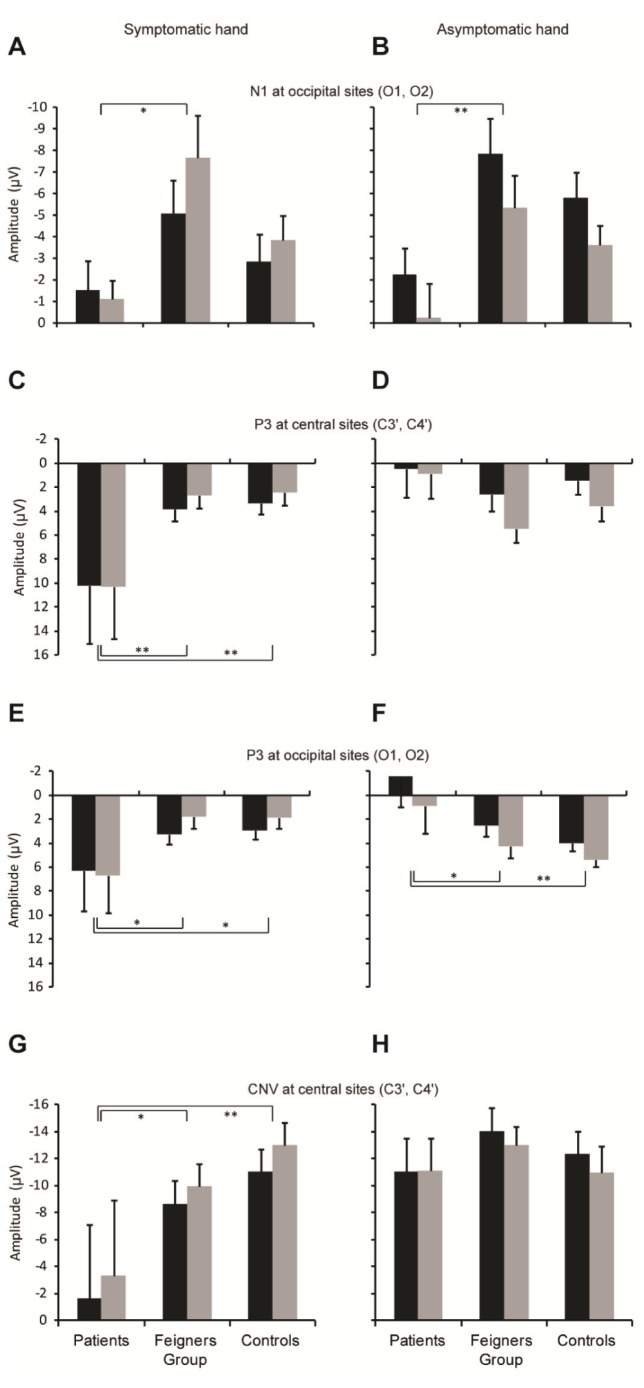
Quantification of ERP measures. (A–B) Mean (+ SEM) occipital N1 amplitude for symptomatic and asymptomatic hand precues respectively. Black bars, left hemisphere; grey bars right hemisphere. (C–D) P3 amplitudes at central electrodes. (E–F) P3 amplitudes at occipital electrodes. (G–H) CNV amplitudes at central electrodes. Negative upwards in all graphs. *P<.05, **P<.01.

In contrast the N1 component recorded at occipital electrodes (O1, O2) was dramatically different across groups, as illustrated in Fig. 2A,B and quantified in Fig. 3A,B. When the precue was on the left, indicating that a movement of the symptomatic (left) hand would be required (Fig. 3A), there was a significant Group by Hemisphere interaction (F_2,24_ = 6.7, *P* = .005), with patients having a smaller N1 amplitude than feigners (*P*<.05), while controls had intermediate N1 amplitudes that were not different from feigners or patients. Further, while both feigners and controls had significantly larger N1 amplitudes in the right compared to left hemisphere (*P* = 0.016 and *P* = 0.002, respectively), there was no between-hemisphere difference in the patients.

When the precue indicated a response by the asymptomatic right hand to a right-side precue (Fig. 3B), there were significant effects of Group (F_2,50_ = 5.7, *P* = .006) and Hemisphere (F_1,26_ = 46.2, *P* = .001), and no interaction. Post-hoc tests confirmed N1 amplitude was smaller for patients compared to feigners (*P* = 0.004) but unlike the symptomatic hand, patients showed no deficit in lateralization. Thus, patients showed reduced N1 amplitude in response to precues, irrespective of the hand indicated by the precue.

In contrast to N1, analysis of P3 amplitude revealed a striking difference for patients specific to the symptomatic hand. When the symptomatic hand was precued, patients, in contrast to both feigners and controls, exhibited a prominent *enhancement* in P3 amplitude at a latency of ∼375 ms (Fig. 2A,C, 3C,E). The enhancement occurred in both hemispheres, at both central and occipital electrodes (Central: Group, F_2,54_ = 8.4, *P* = 0.001; post-hoc tests *P* = 0.002, *P* = 0.001 for patients *vs* feigners and controls. Occipital: Group, F_2,50_ = 4.4, *P* = 0.018; post-hoc tests *P* = 0.031, *P* = 0.025).

As illustrated in Fig. 2A,C, in control and feigning participants preparing to move the left hand, the initial visual ERP components were followed by an expected CNV (Fig. 2A,C). In contrast, while the CNV trajectory for patients appeared to run in parallel to the other groups, its initiation was offset by the much greater P3 amplitude. Quantitative analysis of the last 100 ms of the preparatory period for C3' and C4' (Fig. 3G) confirmed that the patients’ CNV amplitude, relative to baseline, was significantly offset compared to that of feigners and controls (Group, F_2,54_ = 6.4, *P* = 0.003; post-hoc tests *P* = 0.041, *P* = 0.002 for patients *vs* feigners and controls. There was no significant effect of hemisphere on CNV amplitude).

Very different results for P3 and CNV amplitudes were found for precues signaling movement of the right (asymptomatic) hand. Unlike the symptomatic hand, there was no significant increase in P3 amplitude in patients at either central or occipital sites (Fig. 2D, 3D,F,H). In fact, at occipital electrodes when the right hand was precued, the P3 amplitude of patients was significantly *smaller* compared to both other groups (Fig. 2B, 3F; Group, F_2,50_ = 8.6, *P* = 0.001; post-hoc tests *P* = 0.011, *P* = 0.001 for patients *vs* feigners and controls). At both central and occipital electrodes, the subsequent CNV waveforms converged, thus in contrast to the CNV associated with upcoming movement of the symptomatic hand, there were no significant differences in CNV amplitude at the end of the preparatory period.

Additional planned comparisons conducted to specifically examine between-hand differences in P3 and CNV amplitudes, revealed significantly greater P3 amplitudes and significantly reduced CNV amplitudes for precues signaling the patients’ symptomatic (left) hand compared to the asymptomatic (right) hand (P3 Central: F_1,18_ = 7.9, *P* = .011. P3 Occipital: F_1,18_ = 6.3, *P* = .022. CNV: F_1,18_ = 5.1, *P* = .036.). There was no effect of hand on P3 or CNV amplitudes for feigners or controls.

## Discussion

We found strong effects in the amplitude of N1 and P3 ERP components in patients with conversion paresis. In contrast to feigners, patients showed reduced N1 amplitudes for responses to precues indicating either hand. It has been reported that strong focusing of attention to one side can reduce N1 amplitude to stimuli on either side [23], [25], so this bilateral effect could reflect extra attention to precues indicating that they were to move their symptomatic hand. However, this simple explanation seems unlikely because given the identical task requirements, such an attentional bias would also be expected in feigners. Alternatively, it is reported that N1 scales in amplitude according to the level of cognitive “effort” or attention applied to a task [26]. It follows that a parsimonious explanation for the lower N1 amplitudes in patients is that it reflects a generalised reduction in the level of cognitive effort or attentional resources applied to identifying task stimuli. Such an apriori set, affecting responsiveness in a global way, is conceptually similar to Bayesian approaches to understanding conversion disorder, where powerful “priors” or expectations are postulated to override normal sensory processing [27], [28]. Given that the key difference between conversion disorder and feigning concerns conscious awareness of the origin of deficits, it is also relevant that N1 is thought to index brain processes involved in active stimulus discrimination [29] and conscious stimulus perception [30].

In contrast to the side-independent reduction of N1 amplitude, patients showed a strong hand-*specific* modulation of P3 amplitude. For movement of the symptomatic hand, patients had significantly enhanced P3 amplitude following precue onset, whereas for trials involving the asymptomatic hand there was a localized reduction in P3 amplitude. The P3 is thought to reflect sensory processing occurring after initial discrimination of the precue. Experimentally, it is typically evoked by rare stimuli, for example in “oddball” paradigms. Theories for the functional correlates of P3 (for a detailed review see Polich [31]) include that it is involved in context updating when circumstances change, with variations in amplitude reflecting allocation of attention when a change is detected. However in the present study, purely statistical “oddball effects” are unlikely to account for the differences in P3 amplitude seen in patients for one particular precue, because either precue was equally probable. Further, the dramatic P3 modulation in patients is unlikely to just be a necessary concomitant of “preparing to move weakly”, because feigners and patients generated equally deficient movements. It is also unlikely to be a consequence of “practice effects” arising from the more longstanding deficit in patients, because feigners showed no evidence of any practice effect, with no alteration in performance over the course of the 8 blocks of trials. Furthermore, if the feigners were “less” practiced than the patients at presenting with deficient movement, it could be argued the feigners would require *more* allocation of attention to feign, which would be predicted to increase P3 amplitude. Instead, patients appear to have specific differences in the early processing of precues dependent on the information each contains.

In clinical practice the distinction of conversion paresis from malingering or factitious disorder is currently based on judgments of honesty and integrity, and can be challenging [32]. Although the significant differences in N1 and P3 amplitudes between patients and feigners raises the interesting possibility these measures may help to distinguish such populations, further research is required to establish the robustness of differences at the individual level, and whether feigners acting on request are actually representative of the other groups. Moreover, such EEG markers may be specific to particular conversion disorder presentations. For example, the hand-specific modulation of P3 observed in our patient cohort stands in marked contrast to results of studies with a primary focus on sensory conversion disorder, where the P3 amplitudes were either not enhanced or absent[33]–[35]. While this may reflect differences between oddball and movement-precuing paradigms, the possibility of differences between conversion disorder subtypes cannot be ruled out.

There are several possible reasons why precue information might lead to increased neural activity reflected in the enhanced P3 in conversion paresis patients compared to feigners. While both feigners (consciously) and patients (unconsciously) may pay extra attention to precues associated with their symptomatic hand in order to produce the observed differential performance, emotional reactivity may differ. Patients might be expected to attach a higher emotional loading on, or be more threatened or stressed by precues indicating that movement of the symptomatic hand is required. Indeed, conversion paresis patients have been shown to express ERP correlates of hyperactive action monitoring for the symptomatic hand in a stimulus conflict task [36]. It is has been shown that evaluation of threatening or negative stimuli can enhance early P3 amplitude in patients with depression [37] or when the affective stimuli are particularly relevant to individual life histories [38]. Interestingly, an fMRI imaging study of conversion paresis affecting the lower limb found activation in the midline lingual gyrus (extrastriate visual cortex) of patients but not feigning controls [8]. Activation of this region has previously been associated with processing of visual cues during high arousal [39]. However, if the P3 effect in patients is due to an emotional loading, it may index a specifically unconscious form of emotional processing. A key criterion for diagnosis of conversion disorder is that patients are sincerely unconscious of the origin of their symptoms. For this criterion to hold, any threat generated by precues relating to the symptomatic hand must be unconsciously perceived.

Apart from unconscious emotional processing, the enhanced P3 may also relate to the lack of awareness that patients have about the origin of their symptoms. Cojan and colleagues compared changes in regional cerebral blood flow in a conversion paralysis patient with feigning controls participants and found increased activation of midline brain regions in the patient, thought to be associated with processing of self-related information [6]. These changes in blood flow and the electrophysiological changes we report here may represent engagement of an inhibitory system responsible for suppressing awareness of self-agency with respect to one’s own movements. Indeed, other functional imaging studies of patients with conversion tremor found reduced connectivity between multisensory integration limbic regions and sensorimotor cortical regions, including the supplementary motor area, normally necessary for the generation of a sense of self-agency [40], [41]. Consistent with this role for P3, increased P3 amplitudes are seen even in healthy participants when performing tasks specifically designed to disrupt the sense of self-agency [42].
